# Prognostic significance of SOCS1 and SOCS3 tumor suppressors and oncogenic signaling pathway genes in hepatocellular carcinoma

**DOI:** 10.1186/s12885-020-07285-3

**Published:** 2020-08-17

**Authors:** Md Gulam Musawwir Khan, Amit Ghosh, Bhavesh Variya, Madanraj Appiya Santharam, Awais Ullah Ihsan, Sheela Ramanathan, Subburaj Ilangumaran

**Affiliations:** 1 Immunology graduate program, Department of Immunology and Cell Biology, Faculty of Medicine and Health Sciences, University of Sherbrooke, 3001 North 12th avenue, Sherbrooke, QC J1H 5N4 Canada; 2 Cell biology graduate program, Department of Immunology and Cell Biology, Faculty of Medicine and Health Sciences, University of Sherbrooke, 3001 North 12th avenue, Sherbrooke, QC J1H 5N4 Canada; 3 CRCHUS, Sherbrooke, Québec J1H 5N4 Canada

**Keywords:** Hepatocellular carcinoma, SOCS1, SOCS3, TCGA, Tumor suppressor, Oncogenic signalling, Prognosis

## Abstract

**Background:**

*SOCS1* and *SOCS3* genes are considered tumor suppressors in hepatocellular carcinoma (HCC) due to frequent epigenetic repression. Consistent with this notion, mice lacking SOCS1 or SOCS3 show increased susceptibility to diethylnitrosamine (DEN)-induced HCC. As SOCS1 and SOCS3 are important regulators of cytokine and growth factor signaling, their loss could activate oncogenic signaling pathways. Therefore, we examined the correlation between *SOCS1*/S*OCS3* and key oncogenic signaling pathway genes as well as their prognostic significance in HCC.

**Methods:**

The Cancer Genome Atlas dataset on HCC comprising clinical and transcriptomic data was retrieved from the cBioportal platform. The correlation between the expression of *SOCS1* or *SOCS3* and oncogenic pathway genes was evaluated using the GraphPad PRISM software. The inversely correlated genes were assessed for their impact on patient survival using the UALCAN platform and their expression quantified in the regenerating livers and DEN-induced HCC tissues of mice lacking *Socs1* or *Socs3*. Finally, the Cox proportional hazards model was used to evaluate the predictive potential of *SOCS1* and *SOCS3* when combined with the genes of select oncogenic signaling pathways.

**Results:**

*SOCS1* expression was comparable between HCC and adjacent normal tissues, yet higher *SOCS1* expression predicted favorable prognosis. In contrast, *SOCS3* expression was significantly low in HCC, yet it lacked predictive potential. The correlation between *SOCS1* or *SOCS3* expression and key genes of the cell cycle, receptor tyrosine kinase, growth factor and MAPK signaling pathways were mostly positive than negative. Among the negatively correlated genes, only a few showed elevated expression in HCC and predicted survival. Many PI3K pathway genes showed mutual exclusivity with *SOCS1* and/or *SOCS3* and displayed independent predictive ability. Among genes that negatively correlated with *SOCS1* and/or *SOCS3*, only *CDK2* and *AURKA* showed corresponding modulations in the regenerating livers and DEN-induced tumors of hepatocyte-specific *Socs1* or *Socs3* deficient mice and predicted patient survival. The Cox proportional hazards model identified the combinations of *SOCS1* or *SOCS3* with *CXCL8* and *DAB2* as highly predictive.

**Conclusions:**

*SOCS1* expression in HCC has an independent prognostic value whereas *SOCS3* expression does not. The predictive potential of *SOCS1* expression is increased when combined with other oncogenic signaling pathway genes.

## Background

Hepatocellular carcinoma (HCC) remains the fifth most prevalent and the third most lethal cancer worldwide despite significant advances in understanding the molecular pathogenic mechanisms [1, 2]. New therapies targeting various oncogenic signaling pathways are in various phases of development and clinical testing [3]. The availability of mouse genetic models and transcriptomic data from The Cancer Genome Atlas (TCGA) consortium are fueling efforts to identify new therapeutic targets as well as to develop prognostic biomarkers [4–7].

Among the many genes implicated in HCC pathogenesis, the *SOCS1* gene coding of suppressor of cytokine signaling 1 is repressed by epigenetic mechanisms in up to 65% of human primary HCC specimens [8, 9]. The *SOCS3* gene is also repressed in 33% of HCC samples [10]. Cytokines and growth factors regulated by SOCS1 and SOCS3 are important players in both physiologic and neoplastic growth of hepatocytes [11–13]. We and others have studied liver regeneration in mice lacking *Socs1* or *Socs3* and their susceptibility to HCC induced by diethylnitrosamine (DEN) [14–17]. These studies reported an increased rate of liver regeneration and heightened susceptibility to DEN-induced HCC in these mice. These findings, in corroboration with clinical data on epigenetic repression of *SOCS1* and *SOCS3* genes in HCC specimens, clearly established non-overlapping tumor suppressor functions of SOCS1 and SOCS3 in the liver. Moreover, HCC invariably arises in cirrhotic livers, which provides not only an inflammatory environment for hepatocarcinogenesis but also increases the availability of cytokines and growth factors [12]. SOCS1 also regulates the hepatic fibrogenic response by regulating cytokine and growth factor signaling in hepatic stellate cells and in liver resident and infiltrating immune cells [14, 18, 19]. SOCS3 also plays an anti-fibrogenic role in the liver [20]. Therefore, SOCS1 and SOCS3 may regulate hepatocyte proliferation directly as well as indirectly by modulating the liver tissue environment.

SOCS1 and SOCS3 share maximum sequence homology and structural similarity among the SOCS family members, yet significantly differ in their ability to control cytokine and growth factor signaling [21]. Whereas SOCS1 controls hepatocyte growth factor (HGF) signaling via the receptor tyrosine kinase MET [22], SOCS3 is essential to control IL-6 and epidermal growth factor receptor (EGFR) signaling [17]. SOCS1 also regulates the paradoxical oncogenic functions of the cell cycle inhibitor CDKN1A [15]. These findings imply diverse roles for SOCS1 and SOCS3 in regulating hepatocyte proliferation and neoplastic growth. Whether all these functions are compromised in primary HCC is not yet known.

To gain a deeper understanding of the tumor suppressor functions of SOCS1 and SOCS3 in HCC, and to identify the signaling pathways that are aberrantly activated in the absence of SOCS1 or SOCS3, we carried out a systematic analysis on the TCGA dataset on liver HCC (TCGA-LIHC) [6]. We evaluated how the expression of *SOCS1* and *SOCS3* genes correlates with genes implicated in hepatocarcinogenesis, emphasizing genes that regulate hepatocyte proliferation, survival and neoplastic growth. Our findings show that the expression of *SOCS1* and *SOCS3* negatively correlates with several genes in a similar fashion, but also show distinct regulation of some genes in several oncogenic signaling pathways. The latter could explain, at least partly, the inability of SOCS3 to compensate for the loss of SOCS1 and vice versa in animal models of HCC. We identify *SOCS1* but not *SOCS3* as an independent prognostic factor, whereas both display improved predictive potential when combined with certain genes of key oncogenic signaling pathways.

## Methods

### TCGA-LIHC dataset

The gene expression analysis was performed on the RNAseq data from the TCGA provisional dataset on LIHC generated by the TCGA Research Network (https://www.cancer.gov/tcga) [6]. The provisional TCGA-LIHC cohort contains 442 specimens, of which RNAseq V2 data are available for 373 samples. Within this dataset, fifty samples contained paired tumor and adjacent normal tissues. The gene expression dataset was downloaded from the cBioportal suite for cancer genomics research (https://www.cbioportal.org) and analyzed using various publicly available tools as illustrated in the workflow in Supplementary Figure S1.

### Correlation between SOCS1/SOCS3 and oncogenic signaling pathway genes

The various oncogenic signaling pathway genes found to be commonly affected in diverse cancers have been identified and categorized by the TCGA working groups [23]. Among these pathways, those related to cell survival and proliferation were chosen for comparative analysis with *SOCS1* and *SOCS3* genes. These pathways include cell cycle control (34 genes), RTK signaling and angiogenesis (19 genes), other growth/proliferation signaling and telomerase (13 genes), RAS-RAF-MEK-MAPK signaling (26 genes) and PI3K-AKT-MTOR signaling (17 genes). The genes within each pathway are listed in the respective figures. Correlation between the expression of *SOCS1*/*SOCS3* and those of the query genes in the aforementioned oncogenic signaling pathways was evaluated by Pearson’s nonparametric correlation analysis (one-tailed) using the GraphPad Prism (version 8) software. The correlation coefficient *(*ρ-value*)* was represented in a heatmap to reveal the relationship between *SOCS1/SOCS3* and genes within the selected pathways. Statistical significance of the correlation is indicated by asterisks within the heatmap.

### Impact of gene expression on patient survival

Correlation between gene expression and patient survival was analyzed using TCGA Clinical Data Resource (TCGA-CDR) available through the UALCAN platform (http://ualcan.path.uab.edu/index.html) [24, 25]. UALCAN was used to determine the expression of the query genes in tumor vs non-tumor tissues and across the tumor grades, and its relationship to patient survival. The Kaplan-Meier survival plots were generated by comparing the high expression cases (top 25%) with moderate/ low expression (the remaining 75%). Significance of the survival impact in these two groups was measured by log-rank (Mantel-Cox) *p*-values, or by Gehan-Breslow-Wilcox test as indicated.

### Cox proportional hazard model

The expression levels of all genes in the selected oncogenic signaling pathways were dichotomized according to the pre-determined cut-off values of low or high expression (≤25th percentile and ≥ 25th percentile) and the remaining (>75th percentile and < 75th percentile). Each list was combined with the dichotomized lists for *SOCS1* and *SOCS3*, resulting in four different dichotomous combinations (low *SOCS1* + low gene-X versus rest, low *SOCS1* + high gene-X vs rest, high *SOCS1* + low gene-X vs rest, high *SOCS1* + high gene-X vs rest). All possible combinations of *SOCS1* or *SOCS3* with all query genes were entered into a Cox proportional hazards model using the SAS software v9.4 (SAS Institute Inc., Cary, NC). A stepwise selection was used to determine the most predictive combination for patient survival (better or poor survival). The significant effects of the selected combination of variables were then validated with a univariate log-rank test for the query gene. The same procedure was applied to *SOCS3*.

### Mice, partial hepatectomy and DEN-induced HCC

Hepatocyte-specific SOCS1-deficient mice, generated by crossing *Socs1*^*fl/fl*^ mice with albumin-Cre (*Alb*^*Cre*^) mice, have been already described [15]. *Socs3*^*fl/fl*^ mice were purchased from the Jackson laboratories (B6;129S4-*Socs3*^*tm1Ayos/J*^) and hepatocyte-specific SOCS3-deficient mice were generated by crossing them with *Alb*^*Cre*^ mice. All animal experiments were carried out with the approval of the Université de Sherbrooke Ethical committee on animal experimentation (protocol number 226-17B) under the guidelines set by the Canadian Council on Animal care (CCAC). Partial hepatectomy was carried out on 8–10 weeks old mice under isoflurane anesthesia (2% isoflurane mixed with oxygen) as detailed previously [26], and remnant liver tissues were harvested after 24 h. Experimental HCC was induced by the administration of diethyl nitrosamine (DEN) to 2-weeks old male pups as previously described [15]. The mice were euthanized after 8 months and macroscopic liver tumor nodules and adjacent normal tissues were resected. Euthanasia was carried out using CO2 at a 25% flow rate under isoflurane anaesthesia. Small pieces of tissues were immersed in RNAlater (ThermoFisher) and stored at -20 °C for gene expression analysis.

### Quantitative RT-PCR

Total RNA was isolated from liver tissues using RiboZol™ (AMRESCO, Solon, OH). After verifying the RNA quality, the first complementary strand was made from 1 μg total RNA using QuantiTect® reverse transcription kit (Qiagen). RT-PCR for gene expression analysis was carried out using the CFX-96 thermocycler (Bio-Rad, Mississauga, ON) using the primers listed in Supplementary Table S1. All primers showed more than 90% efficiency with a single melting curve. Expression levels of the housekeeping gene were used to calculate fold induction of the specific genes modulated by the absence or presence of SOCS1 or SOCS3.

## Results

### Reduced expression of *SOCS1* but not *SOCS3* correlates with poor patient survival

**Fig. 1:**
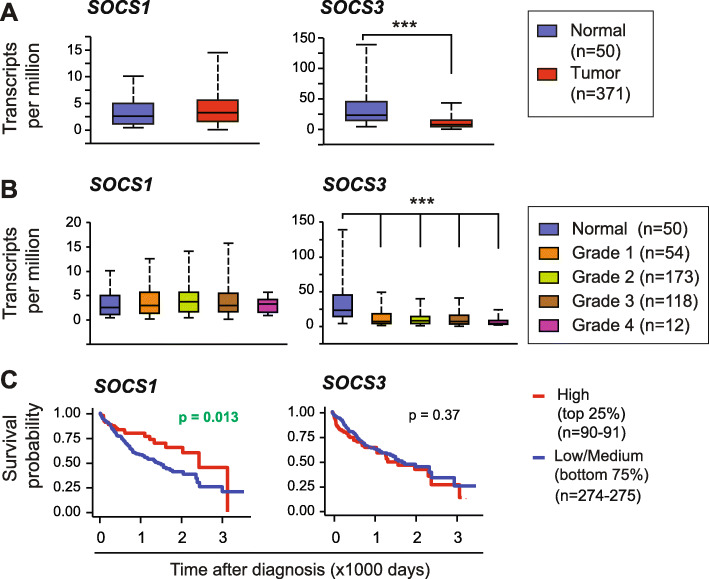
Expression of *SOCS1* and *SOCS3* genes in the TCGA-LIHC dataset and their prognostic significance. **a** Expression levels of *SOCS1* and *SOCS3* genes in the HCC tumors compared to normal liver tissue in the TCGA dataset. **b** Expression levels of *SOCS1* and *SOCS3* in different grades of HCC specimens. **c** Impact of high *SOCS1* and *SOCS3* expression on overall patient survival. The upper high expression quartile was compared with the remaining three-quarts of low/medium expression in the Kaplan-Meier plot

*SOCS1* expression was comparable between tumor tissues and adjacent normal tissues in the TCGA-LIHC dataset, whereas *SOCS3* expression was significantly reduced in tumor tissues (Fig. 1a). *SOCS1* expression was also not significantly affected across tumor grades, whereas *SOCS3* expression was significantly reduced with increasing tumor grade (Fig. 1b). On the other hand, higher *SOCS1* expression correlated positively with overall patient survival, whereas the *SOCS3* expression level did not correlate with disease outcome (Fig. 1c). These data suggest that despite the lack of correlation with tumor stage, reduced *SOCS1* expression in tumor tissues displayed an independent prognostic value, whereas reduced *SOCS3* expression per se does not have a prognostic significance. Nonetheless, compelling evidence for the non-overlapping tumor suppressor functions of SOCS1 and SOCS3 from genetic models [14–17] prompted us to investigate the relationship between the expression levels of *SOCS1* and *SOCS3* and the key signaling pathway genes implicated in carcinogenesis.

### Correlation with cell cycle regulation genes

**Fig. 2:**
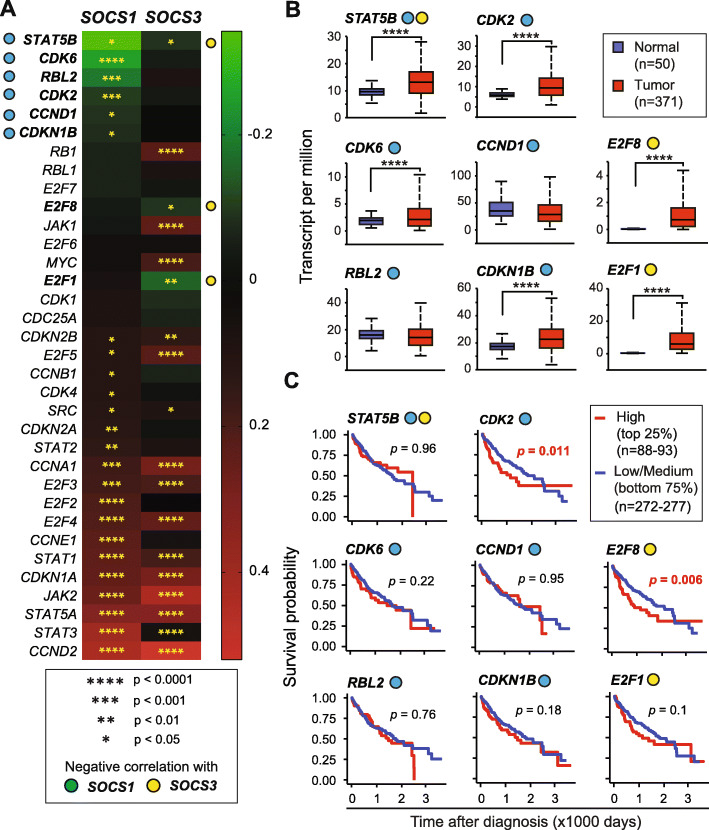
Cell cycle regulation genes predominantly show a positive correlation with *SOCS1* and *SOCS3*. **a** Heatmap showing negative (mutual exclusivity) or positive (co-expression) correlation between cell cycle genes and *SOCS1* or *SOCS3* in the TCGA-LIHC dataset. The extremities of Spearman’s rank correlation coefficient (ρ) are aligned for *SOCS1*, with the color scale shown at right. The ρ-value of − 1 and 1 (green to red) implies a stronger linear relationship of mutual exclusivity and co-occurrence, respectively. Asterisks within the heatmap indicate the statistical significance of the Spearman correlation. Blue circles on the left indicate Genes showing statistically significant negative correlation with SOCS1 and yellow circles on the right mark genes showing mutual exclusivity with SOCS3. **b** Genes that show significant negative correlation with *SOCS1* and/or *SOCS3* were evaluated for their expression levels in HCC tumors compared to normal liver tissues. **c** Prognostic potential of the above genes was evaluated by comparing the upper quartile of high expression against the remaining three-quarts of low/medium expression by Kaplan-Meier plot. For genes showing statistically significant prognostic potential, with high gene expression correlating poor overall survival, the *p*-values are indicated in red-color font

Like in other cancers, the cell cycle pathway genes are frequently altered in HCC [23]. As SOCS1 and SOCS3 are implicated in the regulation of HGF, EGF and IL-6 signaling that promote hepatocyte proliferation and HCC pathogenesis [17, 22, 26, 27], we first evaluated the relationship between the expression of *SOCS1* and *SOCS3* with the cell cycle regulation genes. *SOCS1* showed a significant negative correlation with six of the thirty-four cell cycle genes (*STAT5B*, *CDK6*, *RBL2*, *CDK2*, *CCND1* and *CDKN1B*), whereas *SOCS3* showed mutual exclusivity with only three namely, *STAT5B*, *E2F8*, and *E2F1* (Fig. 2a). Most of these genes (*STAT5B*, *CDK6*, *CDK2*, *CDKN1B*, *E2F8*, and *E2F1*) showed high mRNA expression in tumor tissues compared to adjacent non-tumor tissues (Fig. 2b). However, the elevated expression of most of these genes in HCC tumor tissues did not predict patient survival except *CDK2* and *E2F8,* for which a higher expression was associated with poor survival (Fig. 2c). Surprisingly, many cell cycle genes showed a positive correlation with *SOCS1* and *SOCS3*, eighteen with *SOCS1* and fifteen with *SOCS3* (Fig. 2a)*.* Many of these positively correlated genes displayed the ability to independently predict poor prognosis (Supplementary Table S2). As expected, a strong positive correlation was observed for both *SOCS1* and *SOCS3* with *STAT3* and *STAT5A* (Fig. 2a). In contrast, *STAT5B* displayed a weak mutual exclusivity with both *SOCS1* and *SOCS3*.

### RTK signaling and angiogenesis pathways

**Fig. 3:**
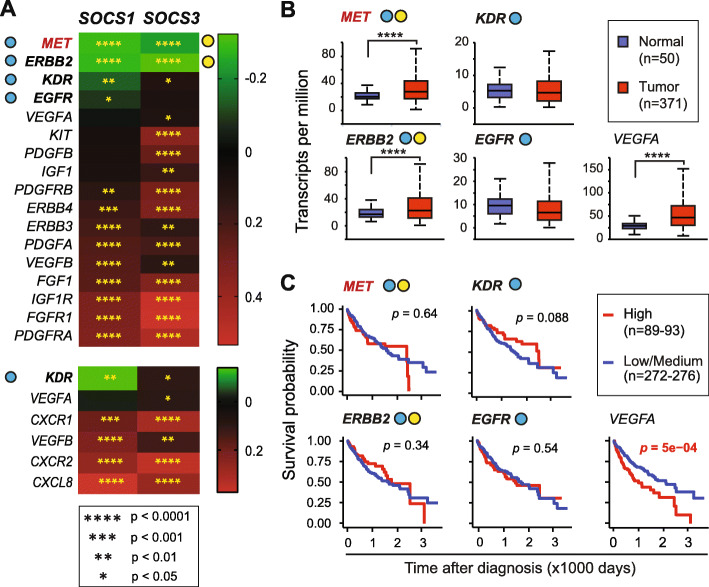
Only a few RTK and angiogenesis genes show a negative correlation with *SOCS1* or *SOCS3*. **a** Correlation between the expression of sixteen RTK signaling and six angiogenesis pathway (three overlapping with the RTK pathway) genes and *SOCS1* or *SOCS3*. MET is not listed in the TCGA oncogenic signaling genes but is included for reasons detailed in the text. **b** Expression levels of genes, which show a significant negative correlation with *SOCS1* and/or *SOCS3,* in HCC tumors and normal liver tissues. **c** Prognostic potential of the above genes was evaluated as described in Fig. 2c

Receptor tyrosine kinase (RTK) signaling activated by growth factors HGF, EGF and IGF, which promote physiologic hepatocyte proliferation, can become oncogenic in transformed cells [13, 28]. Some of the RTKs and certain chemokine receptors promote angiogenesis during tumor growth [29]. Out of the six genes of the angiogenesis pathway, three overlap with the sixteen driver genes of the RTK pathway. Even though the MET RTK is not included in the list of oncogenic RTKs pathway genes, we included MET in our study because deregulated MET signaling promotes HCC, and SOCS1 and SOCS3 are known to regulate MET kinase activity [23, 26, 27]. Among the oncogenic RTK and angiogenesis pathway genes, a significant negative correlation was found only for *ERBB2* (also known as EGFR2, HER2) and *MET* with both *SOCS1* and *SOCS3*, and additionally for *EGFR* with SOCS1 (Fig. 3a). Among these genes, elevated expression in cancer tissues was observed for *MET* and *ERBB2* (Fig. 3b), but neither of them predicted patient survival (Fig. 3c). Among the angiogenesis pathway genes, *KDR* negatively correlated with *SOCS1*, but *SOCS3* showed a positive correlation with *KDR* and *VEGFA* (Fig. 3a). Whereas VEGFA is elevated in HCC and impacts negatively on patient survival, KDR expression was not increased in HCC (Fig. 3b-c). The majority of the RTK and angiogenesis pathway genes showed a significant positive correlation with both *SOCS1* and *SOCS3* (Fig. 3a), and only a few of the RTK/angiogenesis pathway genes (*ERBB3*, *VEGFA* and *CXCL8*) displayed the ability to predict the disease outcome (Supplementary Table S2).

### Other growth factors/proliferation signaling pathways and telomerase maintenance

**Fig. 4:**
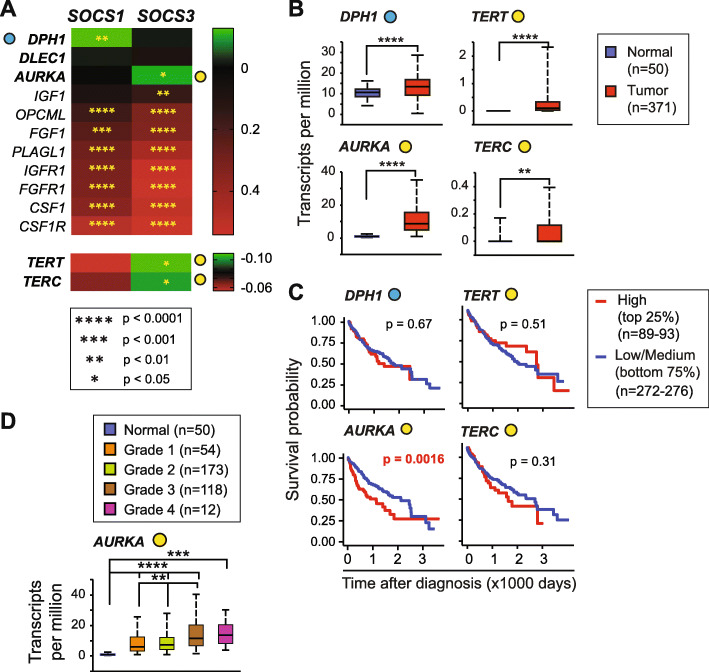
*AURKA* expression negatively correlates with *SOCS3* and predicts poor survival. **a** Correlation between *SOCS1* and *SOCS3* gene expression with other growth signaling pathway genes implicated in oncogenesis. **b** Expression levels of genes, which show a significant negative correlation with *SOCS1* and/or *SOCS3,* in HCC tumors and normal liver tissues. **c** Predictive potential of genes that show a significant negative correlation with *SOCS1* and/or *SOCS3*. **d** Expression of *AURKA* across the tumor grade

Besides the classical growth factor signaling pathways discussed above, certain other growth factors and cell proliferation signals contribute to the pathogenesis of several cancers including HCC. This pathway includes genes coding for colony-stimulating factor-1 (CSF1) and its receptor CSF-1R, fibroblast growth factor (FGF)-FGFR and insulin-like growth factor (IGF)-IGFR systems [30–33], and a select set of less well-studied molecules implicated in carcinogenesis such as aurora kinase (*AURKA*) and diphthamide biosynthesis 1 (DPH1) [34, 35]. A majority of these eleven genes of this group show a positive correlation with *SOCS1* and *SOCS3* in the TCGA HCC dataset (Fig. 4a, Supplementary Table S2). Two key genes involved in telomerase maintenance reverse transcriptase (*TERT*) and the telomerase RNA component (*TERC*), which are critical for telomerase reactivation during HCC pathogenesis [36], are also included within this group. Notably, significant mutual exclusivity was observed for *SOCS3* with *TERT, TERC* and *AURKA*, and for SOCS1 with *DPH1* (Fig. 4a). All these four genes showed higher mRNA expression in HCC tumors compared to normal liver tissue (Fig. 4b). However, among them, only *AURKA* displayed significant predictive potential, with high expression correlating to poor survival (Fig. 4c) and its expression is significantly increased in advanced HCC (Fig. 4d). Even though long telomeres characterize HCC, *TERT* and *TERC* expression levels lacked predictive potential in the TCGA-LIHC dataset.

### RAS-RAF-MAPK signaling pathways

**Fig. 5:**
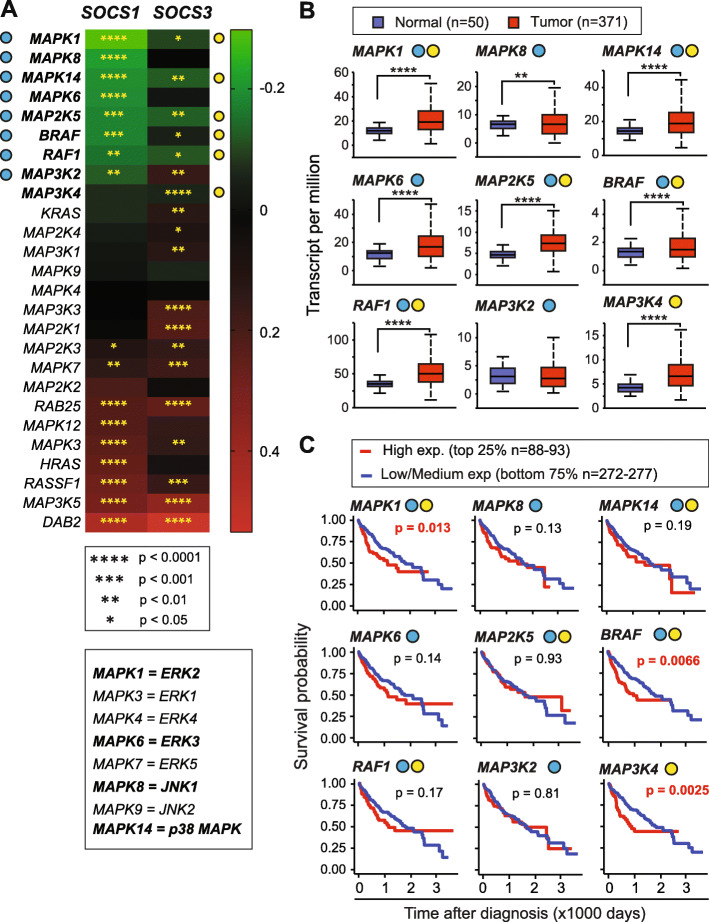
Correlation between *SOCS1*, *SOCS3* and the RAS-MAPK pathway genes and their prognostic significance. **a** The oncogenic RAS-RAF-MEK-MAPK pathway genes were compared with SOCS1 and SOCS3 to assess mutual exclusivity and co-expression. Certain common names of genes in this pathway are indicated below. **b** Expression levels of genes, which show significant negative correlation with *SOCS1* and/or *SOCS3,* in HCC tumors and normal liver tissues. **c** Prognostic potential of the above genes

The mitogen-activated protein kinases (MAPK) contribute to carcinogenesis via promoting many cellular functions such as cell survival, proliferation and epithelial to mesenchymal transition [37]. This pathway includes extracellular signal-regulated kinase (ERK), c-Jun N-terminal kinase (JNK) and p38 stress-activated kinase (SAPK), of which ERKs activated downstream of growth factor RTKs, and JNKs activated by inflammatory stimuli are strongly implicated in HCC pathogenesis. The canonical MAPK pathway involves activation of the RAS GTPase and RAF kinases, and then sequential activation of MAP 3 K and MAP 2 K kinases leading to MAPK activation. Activating mutations of *RAS* and *RAF*, and inactivation/repression of endogenous regulators of RAS such as *RASSF1* and *DAB2* are common in many cancers including HCC [38]. Of the twenty-six oncogenic drivers of this pathway, *SOCS1* showed strong mutual exclusivity with eight genes including *RAF1*, *BRAF*, *MAP 2 K5, MAP 3 K2, MAPK1* (ERK2), *MAPK6* (ERK3), *MAPK8* (JNK1) and *MAPK14* (p38 SAPK), some of which also showed a negative correlation with *SOCS3* (Fig. 5a). Even though many of these negatively correlated genes are highly expressed in HCC (Fig. 5b), only *MAPK1*, *BRAF* and *MAP 3 K4* demonstrated the ability to predict patient survival (Fig. 5c). Intriguingly, *SOCS1* showed a strong positive correlation with *HRAS* and *SOCS3* with *KRAS,* and both with *RASSF1, DAB2* and *MAPK3* (ERK1) (Fig. 5a).

### PI3K-AKT-MTOR signaling pathway

**Fig. 6:**
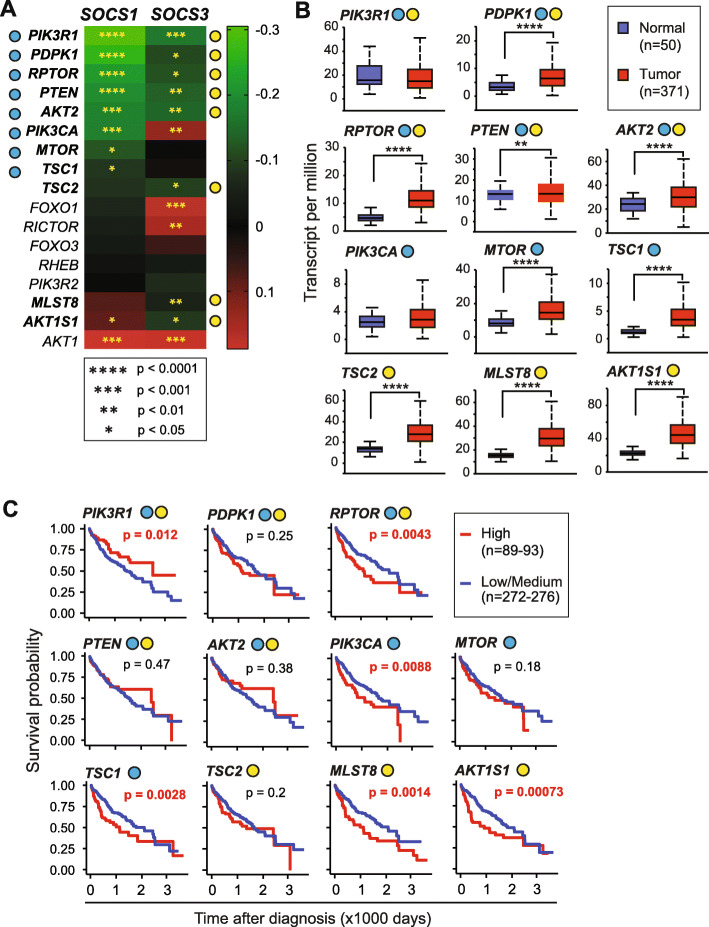
PI3K-AKT pathway genes: relationship to *SOCS1* and *SOCS3* genes and predictive value. **a** Correlation between *SOCS1* and *SOCS3* gene expression with thePI3K-AKT-MTOR signaling pathway genes implicated in oncogenesis. **b** Expression levels of genes, which show a significant negative correlation with *SOCS1* and/or *SOCS3,* in HCC tumors and normal liver tissues. **c** Predictive value of the above genes. Note that the high expression of *PIK3R1* predicts better prognosis

**Fig. 7:**
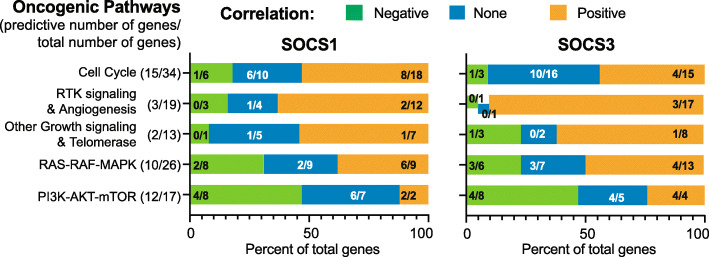
Summary of the correlation between *SOCS1, SOCS3* and the oncogenic signaling pathway genes. Numbers next to the oncogenic signaling pathways indicated on the Y-axis represent those with independent prediction potential over the total number of genes. The X-axis represents the percentage of genes within each pathway that shows negative, no or positive correlation with *SOCS1* or *SOCS3* as a cumulative bar graph. Fractions within the bar graphs represent the number of genes with independent prognostic value out of the total showing negative, no or positive correlation with *SOCS1* or *SOCS3*

The PI3K-AKT-MTOR pathway, which is activated downstream of growth factor and cytokine signaling, is deregulated in multiple cancers including HCC and is considered an important target for therapy [39, 40]. Among the 17 genes of this pathway, five showed a negative correlation with both *SOCS1* and *SOCS3* (*PIK3R1, PDPK1, RPTOR, PTEN, AKT2*), with *PIK3R1* showing the strongest mutual exclusivity (Fig. 6a). Additionally, *TSC1, MTOR*, and *PIK3CA* revealed a negative correlation only with *SOCS1* whereas *AKT1S1*, *TSC2*, and *MLST8* showed mutual exclusivity only with *SOCS3*, making PI3K-AKT-MTOR pathway the most closely related to *SOCS1/SOCS3* (Fig. 7). Notably, *PIK3CA* (the catalytic subunit of PI3K) showed mutual exclusivity with *SOCS1* but co-occurrence with *SOCS3*, whereas the AKT target *AKT1S1* showed an inverse relationship (Fig. 6a). Among the eleven PI3K-AKT-MTOR pathway genes negatively correlated with *SOCS1/SOCS3*, all except *PIK3CA* and *PIK3R1* showed significantly elevated expression in HCC tumors compared to normal liver tissue (Fig. 6b), and several of them also displayed significant independent predictive value, with high expression associated with poor survival (Fig. 6c). Intriguingly, elevated expression of *PIK3R1*, which showed a negative correlation with *SOCS1* and *SOCS3*, was associated with a better disease outcome (Fig. 6c).

As SOCS1 and SOCS3 are tumor suppressors implicated in regulating cytokine and growth factor signaling pathways, we expected a predominantly inverse correlation between *SOCS1/SOCS3* and oncogenic signaling pathway genes implicated in HCC. However, this was only observed within the PI3K-AKT-MTOR pathway (Fig. 7; Supplementary Table S2). Among the genes that showed a negative correlation with *SOCS1* and/or *SOCS3*, nine genes with high expression in tumor tissues predicted poor prognosis, whereas low expression of *PIK3R1* was associated with bad prognosis (Table 1).

**Table 1:** Genes that negatively correlate with *SOCS1* and/or *SOCS3* and their impact on patient survival. * High expression predicts better survival, for all others poor survival

SOCS	Negatively correlated genes	Oncogenic signaling Pathway	Upregulation in tumor vs normal *p* value	Survival Probability *p* value
***SOCS1***	*–*	–	No difference	0.013
***SOCS1***	*CDK2*	Cell cycle regulation	0.0001	0.011
	PIK3R1*	PI3K-AKT-MTOR	No difference	(0.012)*
***SOCS3***	*E2F8*	Cell cycle regulation	0.0001	0.006
	*AKT1S1*	PI3K-AKT-MTOR	0.0001	0.00073
	*MLST8*	PI3K-AKT-MTOR	0.0001	0.0014
	*AURKA*	Other Growth signaling	0.0001	0.0016
	*MAP 3 K4*	RAS-RAF-MEK-MAPK	0.0001	0.0025
***SOCS1,*** ***SOCS3***	*RPTOR*	PI3K-AKT-MTOR	0.0001	0.0043
	*BRAF*	RAS-RAF-MEK-MAPK	0.0001	0.0066
	*MAPK1*	RAS-RAF-MEK-MAPK	0.0001	0.013

*High expression predicts better survival, for all others poor survival

### Validation of genes that inversely correlated to *SOCS1* or *SOCS3* expression

**Fig. 8:**
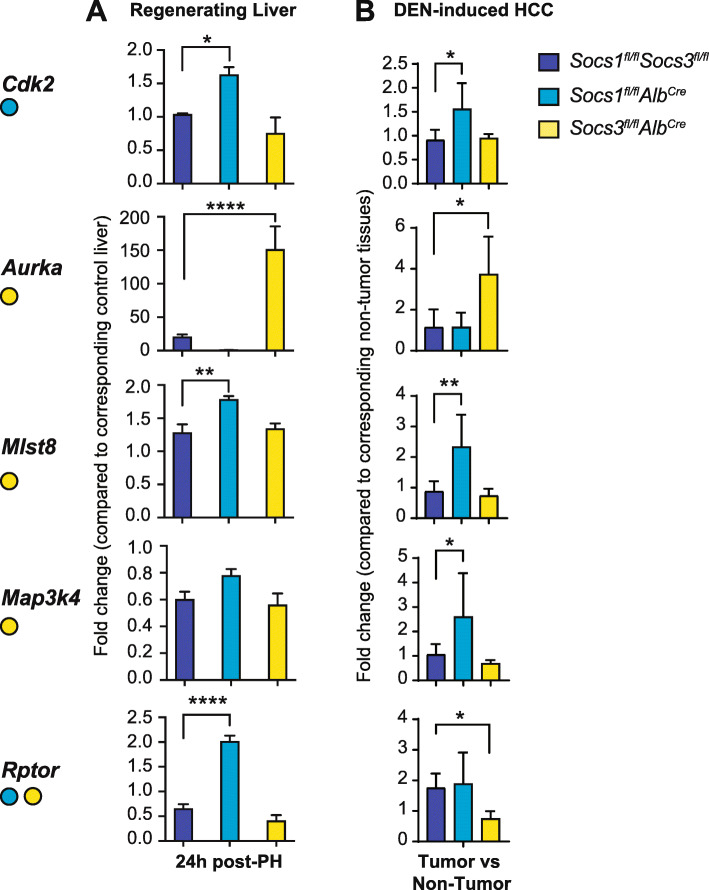
Validation of genes that negatively correlate with *SOCS1* or *SOCS3* in murine models. **a** Partial hepatectomy was carried out on 8–10 weeks old mice lacking *Socs1* or *Socs3* in hepatocytes and control mice. The expression of the indicated genes in the regenerating livers was evaluated 24 h later by qRT-PCR. *n* = 4–6 mice per group. **b** Mice lacking *Socs1* or *Socs3* in hepatocytes and control mice were treated with DEN (25 mg/kg body weight) at 2 weeks of age and livers collected at 8–10 months of age. Tumor nodules and adjacent normal liver tissues were resected and expression of the indicated genes was evaluated by qRT-PCR. *n* = 4–6 mice per group. *p*-values were calculated by one-way ANOVA along with Tukey’s Multiple Comparison test: * *p* < 0.05, ** *p* < 0.01, *** *p* < 0.001, **** *p* < 0.0001

Next, we used mice lacking SOCS1 or SOCS3 in hepatocytes to validate key oncogenic signaling pathway genes that negatively correlated with *SOCS1* or *SOCS3* in the TCGA-LIHC dataset. Physiological hepatocyte proliferation was induced in *Socs1*^*fl/fl*^*Alb*^*Cre*^, *Socs3*^*fl/fl*^*Alb*^*Cre*^ and *Socs1*^*fl/fl*^*Socs3*^*fl/fl*^ control mice by partial hepatectomy (PH), and gene expression was evaluated in regenerating livers 24 h later (Fig. 8a). To study gene expression associated with pathological hepatocyte proliferation, HCC was induced in hepatocyte-specific SOCS1- or SOCS3- deficient mice using the hepatocarcinogen DEN (Fig. 8b). Liver tumor nodules and adjacent non-tumor tissues, obtained 8–10 months after DEN injection, were evaluated for gene expression.

*Cdk2*, which is negatively correlated with *SOCS1* expression in the TCGA dataset, was significantly upregulated in SOCS1-deficient liver following PH as well as in DEN-induced HCC (Fig. 8a-b). Similarly, *Aurka*, which is negatively correlated with *SOCS3* in the TCGA dataset, was upregulated more than 100-fold in SOCS3-deficient, but not in SOCS1-deficient liver, following PH and in DEN-induced HCC (Fig. 8a-b). In contrast to *Aurka*, *Mlst8* and *Map 3 k4*, which are negatively correlated with *SOCS3* in the TCGA dataset, were not affected by SOCS3 deficiency in the regenerating livers or in HCC tissues, although discernible upregulation of *Mlst8* was observed in the absence of SOCS1 (Fig. 8a-b). *RPTOR*, which is negatively correlated with both *SOCS1* and *SOCS3* in the TCGA dataset, was increased in the regenerating livers of mice lacking SOCS1 in hepatocytes but not in SOCS3-deficient livers. These results show that some of the negative correlations between *SOCS1* or *SOCS3* and the oncogenic signaling pathway genes, notably *CDK2* and *AURKA,* observed in the TCGA dataset are recapitulated in SOCS1- and SOCS3- deficient livers.

**Fig. 9:**
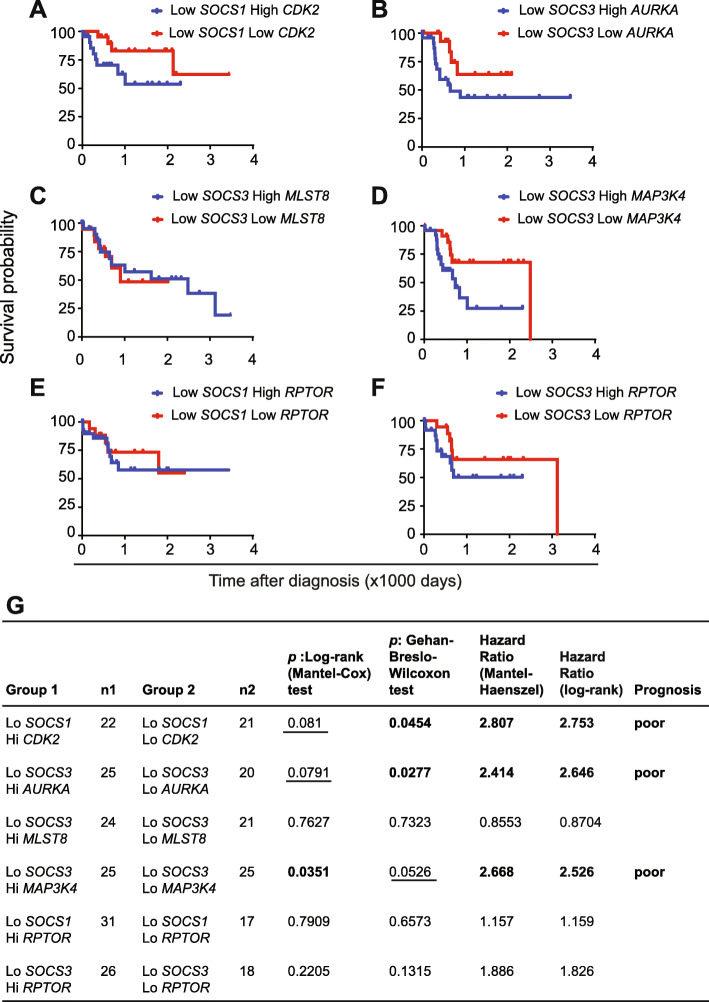
Predictive potential of combining low *SOCS1* or *SOCS3* expression along with high expression of oncogenic pathway genes in the TCGA dataset. **a**-**f** Specimens in the TCGA-LIHC dataset with low *SOCS1* or *SOCS3* expression were segregated into those displaying high or low expression of *CDK2*, *AURKA*, *MLST8*, *MAP 3 K4* or *RPTOR* and compared for patient survival by Kaplan-Meier plot. **g** The number of specimens within each group, the *p*-values calculated by the log-rank and Gehan-Breslow-Wilcoxon tests, the hazard ratios and prognostic potential are shown

Next we evaluated the predictive potential of *SOCS1* and *SOCS3* when combined with the high expression of candidate genes from each oncogenic signaling pathway that showed a negative correlation. As shown in Fig. 9, TCGA-LIHC specimens displaying low *SOCS1*/high *CDK2,* low *SOCS3*/high *AURKA* and low *SOCS3*/high *MAP 3 K4* expression displayed poor prognosis with a hazard ration of more than 2.5 compared to low *SOCS1*/low *CDK2,* low *SOCS3*/low *AURKA* and low *SOCS3*/low *MAP 3 K4* groups, respectively. On the other hand, the predictive potential of high *MLST8* and *RPTOR* expression was not observed within low *SOCS1* or low *SOCS3* groups.

### Impact of oncogenic signaling genes on the predictive potential of *SOCS1* and *SOCS3*

Next, we used the Cox proportional hazards model to evaluate the predictive potential of *SOCS1* and *SOCS3* when combined with oncogenic signaling pathway genes. Even though a high expression of many oncogenic signaling pathway genes independently predicted poor survival (Supplementary Table S2) and some of them showed a better prognostic potential when combined with *SOCS1*/*SOCS3* (*CDK2*, *AURKA* and *MAP 3 K4*; Fig. 9), the Cox model revealed a different set of genes with prognostic potential (Table 2, Supplementary Table S3). Notably, low *SOCS1* displayed a significant predictive potential in combination with *CDK1, CXCL8, CSF1, DAB2* and *TSC1*, with *CXCL8* and *DAB2* showing the highest hazards ratio. *CXCL8* and *DAB2* also predicted poor survival in combination with low *SOCS3*, even though the latter did not display independent predictive ability. A limitation of this analysis is that some combination groups, especially low *SOCS1* or *SOCS3* and high *CXCL8*, had only a very few cases out of a total of 362 cases. In contrast to low expression, high *SOCS1* levels showed limited synergy with most other genes in predicting better survival (Supplementary Table S3). Notably, high *SOCS1* even lost its favorable prognostic value in tumors with high *E2F7*, which independently predicts poor survival.

**Table 2:** Predictive potential of *SOCS1*/*SOCS3* and oncogenic signalling pathway in the Cox proportional hazard model

**Selected gene Combinations**	**Oncogenic signaling Pathway**	**Multivariate Cox model *p*-value**	**Univariate log-rank** ***p*-value**	**Survival probability**	**Number of subjects**	**HR [95% confidence intervals]**
Low *SOCS1* + High *CDK1*	Cell cycle	0.0152	0.0153	Poor	17	2.56 [1.28–5.11]
Low *SOCS1* + **High** ***CXCL8***	RTK signalling, angiogenesis	< 0.0001	< 0.0001	Poor	4	**7.97 [2.85–22.25]**
Low *SOCS1* + High *IGF1R*^a^	RTK signalling, angiogenesis	0.0051	0.007	Poor	6	5.98 [2.09–17.13]
Low *SOCS1* + High *CSF1*	Proliferation	0.0042	0.0042	Poor	11	2.62 [1.19–5.78]
Low *SOCS1* + **High** ***DAB2***	MAPK pathway	< 0.0001	< 0.0001	Poor	8	**7.73 [3.45–17.35]**
Low *SOCS1* + Low *PIK3R1*	PI3K-AKT pathway	0.0102	0.0102	Poor	9	3.52 [1.55–8.01]
Low *SOCS1* + High *TSC1*	PI3K-AKT pathway	0.0248	0.0274	Poor	24	2.84 [1.50–5.38]
Low *SOCS1* + High *RAF1*^a^	MAPK pathway	0.0046	0.0291	Better	28	0.281 [0.11–0.71]
Low *SOCS1* + High *AKT1S1*	PI3K-AKT pathway	0.0034	0.0187	Better	15	0.08 [0.02–0.36]
Low *SOCS1* + Low *PIK3R2*^b^	PI3K-AKT pathway	0.0192	0.0344	Better	28	0.224 [.08–0.65]
Low *SOCS3*^a^ + High *RBL1*	Cell cycle	0.0053	0.0123	Poor	23	2.20 [1.20–4.03]
Low *SOCS3*^a^ + **High** ***CXCL8***	RTK signalling, angiogenesis	0.0017	0.0017	Poor	3	**7.03 [1.70–28.99]**
Low *SOCS3*^a^ + High *FGFR1*^a^	Proliferation	0.0125	0.0173	Poor	4	4.15 [1.31–13.17]
Low *SOCS3*^a^ + Low *DLEC1*^a^	Proliferation	0.0163	0.0324	Poor	22	2.01 [1.12–3.58]
Low *SOCS3*^a^ + **High** ***DAB2***	MAPK pathway	0.0023	0.0023	Poor	10	**3.42 [1.49–7.84]**
Low *SOCS3*^a^ + High *PIK3R1*^b^	PI3K-AKT pathway	0.0232	0.0232	Better	27	0.21 [0.07–0.6]

All possible combinations of *SOCS1* or *SOCS3* with all query genes were analysed in the Cox proportional hazards model as described in materials and methods. High or low expression of individual genes have poor prognosis in univariate analysis unless indicated otherwise: ^a^No prognostic value; ^b^Good prognosis. Genes in bold face show synergy with both low *SOCS1* and low *SOCS3*

Even though high *CXCL8* and *DAB2* transcript levels synergized with low *SOCS1* or *SOCS3* expression to predict high hazards ratio in the Cox model (Table 2), *CXCL8* and *DAB2* showed positive correlation with *SOCS1* and *SOCS3* in the TCGA-LIHC dataset (Figs. 3 and 5) and their high expression independently predicted poor prognosis (Supplementary Table S2) whereas high SOCS1 expression predicted favorable outcome (Fig. 1). Therefore, we examined whether SOCS1 and SOCS3 influenced the expression of *CXCL8* and *DAB2* genes in the mouse models of liver regeneration and DEN-induced HCC. As the *CXCL8* (IL-8) gene is absent in rodents, we examined the genes coding for mouse chemokines KC (*Cxcl1*), MIP-2 (*Cxcl2*) and LIX (*Cxcl2*), which are considered the functional equivalents of human CXCL8 in promoting neutrophil migration [41]. As shown in Supplementary Figure S2, *Cxcl1, Cxcl2* and *Cxcl5* genes showed significantly elevated expression in the regenerating livers of mice lacking SOCS3 or SOCS1, whereas *Cxcl1* was upregulated and *Cxcl2* and *Cxcl5* were downregulated in DEN-induced HCC tissues of both mice compared to control mice. The expression of *Dab2* was not affected by the loss of either SOCS1 or SOCS3 in the mouse liver undergoing physiological or pathological hepatocyte proliferation. These findings suggest (i) the positive correlations between *CXCL8* or *DAB2* and *SOCS1* or *SOCS3* observed in the TCGA-LIHC dataset likely results from deregulated signaling pathways in the tumor tissues and (ii) the elevated *CXCL8* expression worsens the prognosis of cases with low *SOCS1* or *SOCS3* expression as CXCL8 is implicated in promoting tumor angiogenesis [42].

## Discussion

Our study has revealed notable differences in the prognostic utility of *SOCS* gene expression compared to epigenetic repression. Specifically, methylation of the *SOCS1* gene, which occurs in up to 65% of HCC specimens [8, 9], is not reflected in *SOCS1* mRNA expression within the TCGA dataset. On the other hand, the *SOCS3* gene, reported to be repressed only in 33% of HCC cases [10], showed reduced expression in the TCGA dataset. Whereas methylation data based on a positive PCR product reflects the repressed status of the gene in hepatocytes, it is possible that induction of the *SOCS* gene expression in liver-resident and infiltrating immune cells and hepatic stromal cells by a myriad of cytokines and growth factors might contribute to the overall *SOCS1* transcript levels in the TCGA dataset. In support of this possibility, *SOCS1* mRNA expression positively correlated with *CD247* (CD3 zeta chain, all T cells), *CD8A* (CD8^+^ T cells), *NCAM1* (CD56, NK cells) and *IFNG* (activated T and NK cells) (data not shown). Nonetheless, even though *SOCS1* mRNA expression was not significantly different between tumor and normal tissues, higher transcript levels strongly correlated with patient survival (Fig. 1c), highlighting the potential prognostic utility of *SOCS1* expression in HCC.

The positive correlations in the expression of *SOCS1*/*SOCS3* and the oncogenic signaling pathway genes may result from the induction of intact *SOCS1* and *SOCS3* genes as part of the negative feedback regulatory mechanisms to control oncogenic signaling. On the other hand, the negative correlations could arise either from increased oncogenic signaling due to reduced *SOCS1/SOCS3* expression, as well as from reduced oncogenic signaling due to increased *SOCS1/SOCS3* expression. Moreover, SOCS1 and SOCS3 might target certain transcriptional activators or repressors as substrate specific adaptors in ubiquitin-mediated proteasomal degradation [43]. Thus, the loss of SOCS1 or SOCS3 may also affect the oncogenic pathway gene expression indirectly.

As in other cancers, uncontrolled cell cycle progression is a key feature of HCC that results from aberrant expression of cell cycle proteins and/or their regulators [44]. Under conditions of increased cyclin D1 availability, for example from increased growth factor signaling, CDK2 promotes hepatocyte proliferation [45]. CDK2 is a potential therapeutic target in many cancers including HCC [46]. While high *CDK2* expression, either alone or along with low SOCS1 expression, correlated with poor survival in our study (Fig. 2c, Fig. 8c), Sonntag et al., [47] did not find significant prognostic value for *CDK2*, possibly because the latter used the median value to separate the low and high expression groups, whereas in our study we compared the high one-quartile group and the remaining with low/medium expression.

RTK signaling in hepatocytes and endothelial cells is a key promoter of HCC pathogenesis [28, 48]. Indeed, deregulation of this pathway by genomic alterations is over-represented in the TCGA-LIHC dataset, and drugs targeting this pathway such as Sorafenib and Regorafenib are already being used or in advanced clinical trials [2, 6, 48]. However, it is widely perceived that a drug choice based on biomarker analysis could improve the treatment outcome. Key RTK signaling/angiogenesis genes *MET*, *ERBB2*, *KDR* and *EGFR,* all implicated in HCC [49–52], negatively correlate with *SOCS1*, and the first two with *SOCS3* as well. Deregulated EGFR and KDR signaling can contribute to Sorafenib resistance in advanced HCC [51, 53]. However, none of these four genes were able to independently predict patient survival within the TCGA-LIHC dataset (Fig. 3c). This notion is supported by the failure of MET-targeting therapeutics to improve survival outcome that has been recently attributed, at least partly, to the ability of kinase-inhibited MET to promote cell survival [54]. Even though *MET* expression alone was not predictive, ERBB3, which contributes to the resistance to MET inhibition [55] displayed a high predictive potential (Supplementary Table S2). Similarly, even though KDR was not predictive, its ligand *VEGFA* showed a very strong predictive potential (Fig. 3c). These findings identify *ERBB3* and *VEGFA* as potential biomarkers for targeted therapies. CXCL8 (IL-8), a chemokine secreted by inflammatory cells including activated HSCs, induces angiogenic growth factors such as VEGFA in HCC cells and promotes angiogenesis [56]. Strikingly, *CXCL8*, which shows a strong negative prognosis in HCC, displayed marked synergy with low *SOCS1* or *SOCS3* in multivariate analysis (Table 2), suggesting the potential use of these markers together. Indeed, CXCL8 receptor (CXCR1, CXCR2) antagonists [57] could be an important addition to targeted therapeutics in HCC.

The only gene within the other proliferation signaling pathway that showed prognostic potential was *AURKA*, which is a biomarker for cancer development and progression, and a potential target for therapy in HCC [58, 59]. *AURKA* expression is dramatically high in TCGA-LIHC dataset, with a significantly increased expression as the disease progresses, and displays a strong predictive ability for disease outcome either alone or along with *SOCS3* (Fig. 4b-d, Fig. 8c; Table 1). The negative correlation between *AURKA* and *SOCS3* is also highlighted by a more than 100-fold increase in *Aurka* expression in the regenerating livers of hepatocyte-specific SOCS3-deficient mice, and significant upregulation of this gene in DEN-induced HCC in these mice (Fig. 8a-b). One possible mechanism by which *SOCS3* could modulate *AURKA* expression could be via p53, which represses *AURKA* [60]. SOCS3 can promote transcriptional activation of p53 [20]. Whether SOCS3 can also modulate the repressive function of p53 is not known. It is noteworthy that SOCS1, which was shown to activate p53 earlier [61], did not correlate with *AURKA* expression in the TCGA dataset. Clearly, further studies are needed to elucidate the mutual exclusivity of *SOCS3* and *AURKA* expression in HCC.

The RAS-RAF-MAPK pathway is frequently perturbed in HCC and thus is an important therapeutic target [62]. Indeed, the RAF kinase is a key target of Sorafenib that is already used in HCC therapy. Immunohistochemical analysis of HCC specimens in a Chinese cohort revealed a prognostic value for RAF1 [63]. Even though RAF1 expression is elevated in the TCGA-LIHC dataset, it did not have prognostic potential. On the other hand, *BRAF1* and *MAP 3 K4* (MEKK4), which are inversely correlated to *SOCS3* expression, was found to be upregulated in TCGA-LIHC and displayed a high predictive potential. It is noteworthy that *BRAF*, which also negatively correlates with *SOCS1*, has been previously reported to be commonly found in cholangiocarcinoma but not in HCC [64]. The negative regulators of the RAS-RAF-MAPK pathway, which inhibit RAS activity (RASSF1A, RASSF2A, RASSF5, RASAL1) or inhibit the RAF kinase (SPRED1, SPRED2) are frequently repressed by promoter methylation in HCC [38, 65]. DAB2, which attenuates the RAS activation downstream of RTK signaling, is also repressed by promoter methylation [66]. *RASSF1A* and *DAB2* showed coordinate regulation with *SOCS1* and *SOCS3*. As promoter hypermethylation also represses *SOCS1* and *SOCS3*, it is possible that epigenetic repression of both *SOCS* genes as well as the endogenous negative regulators the RAS-RAF-MAPK pathway likely contributes to their coordinate regulation that amplifies the proliferation and anti-apoptotic functions of this pathway, contributing to HCC pathogenesis.

Surprisingly, high expression of *RASSF1* and *DAB2* predicted poor survival in the TCGA-HCC dataset (Supplementary Table S2), instead of a better prognosis expected of their function as negative regulators of the RAS-MAPK pathway. High *DAB2* expression within low *SOCS1* or *SOCS3* expressing subgroups also predicted poor overall survival in the multivariate analysis (Table 2). The reason for this apparent discrepancy is unclear. It is possible that the upregulation of *RASSF1* and *DAB2* may result from mutations that disrupt the normal functions of these tumor suppressors, as in the case of mutant p53, which is highly expressed in many cancers [67]. However, only a negligible proportion of cases in the TCGA dataset revealed mutations for *RASSF1* or *DAB2* (data not shown). It is equally possible that their increased expression could result from a compensatory increase in response to the increased activity of this pathway or mutations in their target proteins. Clearly further studies are needed to resolve this conundrum.

The MTOR pathway is frequently activated in HCC and is associated with poor prognosis [40]. Our findings reveal that out of eleven driver genes of the PI3K-AKT-MTOR pathway analyzed, *RPTOR, PIK3CA, TSC1, MLST8* and *AKT1S1* showed a pronounced negative impact on patient survival whereas *PIK3R1* showed favorable impact (Fig. 6c), raising the possibility of using these genes as prognostic markers. Both SOCS1 and SOCS3 have been implicated in regulating the PI3K-AKT pathway upstream of MTOR. By their ability to promote ubiquitination and proteasomal degradation of insulin receptor substrates 1 and 2 (IRS1, IRS2), which link RTK signaling to PI3K, SOCS1 and SOCS3 can regulate AKT activation in the context of insulin resistance in the liver and other organs [68, 69]. We have shown that SOCS1-deficient primary hepatocytes show increased AKT activation in response to HGF [26]. Our findings show that the expression of both *SOCS1* and *SOCS3* shows a high degree of mutual exclusivity with *PIK3R1* (Fig. 6a). Even though this gene codes for the p85 regulatory subunit of PI3K, there is strong evidence indicating that PIK3R1 also functions as a tumor suppressor by modulating PTEN, AKT and STAT3 [70–72]. Consistent with this role, high *PIK3R1* predicts favorable survival in our analysis (Table 1). Given the tumor suppressor functions and overlapping mechanisms of action of SOCS1, SOCS3 and PIK3R1, further work is needed to disentangle the highly significant negative correlation between *SOCS1/SOCS3* and *PIK3R1*.

## Conclusions

Our findings show that *SOCS1* gene expression in HCC has a significant prognostic value that is further improved when combined with other markers, although studies in other cohorts are needed to confirm these findings. We observed coordinated expression of several oncogenic signaling pathway genes and *SOCS1/SOCS3*, presumably reflecting activation of negative feedback loops. However, nearly half of the PI3K-AKT-MTOR pathway genes showed mutual exclusivity with *SOCS1/SOCS3*, suggesting the loss of SOCS-dependent regulation of RTKs contributing to the increased activity of this signaling pathway. Finally, our study identified at least three genes, *RASSF1* and *DAB2* in the RAS- MAPK pathway and *PIK3R1* in the PI3K-AKT pathway that showed a predictive value opposite of their expected functions, which warrant further investigations. Collectively, *SOCS1* and certain key genes of the oncogenic signaling pathways that show high predictive value in this study could be developed further as combination biomarkers for patient-oriented precision therapeutics in HCC.

## Supplementary information

**Additional file 1: Figure S1.** Workflow of this study. **Figure S2.** Expression of oncogenic signaling pathway genes that synergize with *SOCS1* or *SOCS3* in predicting prognosis by the Cox proportional harzards model in the murine models. *DAB2* and *CXCL8* synergize with *SOCS1* or *SOCS3* in predicting prognosis by the Cox proportional harzards model (shown in Table 2). As the *CXCL8* (IL-8) gene is not present in the mouse, we examined the genes coding for mouse chemokines KC (*Cxcl1*), MIP-2 (*Cxcl2*) and LIX (*Cxcl2*), which are considered the functional equivalent of human CXCL8 in promoting neutrophil migration.(A) Partial hepatectomy was carried out on 8–10 weeks old mice lacking *Socs1* or *Socs3* in hepatocytes and control mice. The expression of the indicated genes in the regenerating livers was evaluated 24 h later by qRT-PCR. *n* = 4–6 mice per group. (B) Mice lacking *Socs1* or *Socs3* in hepatocytes and control mice were treated with DEN (25 mg/kg body weight) at 2 weeks of age and livers collected at 8–10 months of age. Tumor nodules and adjacent normal liver tissues were resected and expression of the indicated genes was evaluated by qRT-PCR. *n* = 4–6 mice per group. *p*-values were calculated by one-way ANOVA along with Tukey’s Multiple Comparison test: * *p* < 0.0001. **Table S1.** List of qRT-PCR primers used in this study. **Table S2.** Impact of the expression of oncogenic pathway genes on survival probability in the TCGA-LIHC dataset. **Table S3.** Combinations of high *SOCS1* or high *SOCS3* and oncogenic signalling pathway genes that show significant prognosis in the Cox proportional hazard model.

## Data Availability

The TCGA-LIHC datasets analysed in the current study are available through the cBioportal suite for cancer genomics research (https://www.cbioportal.org).

## Abbreviations

DEN: Diethyl nitrosamine
HCC: Hepatocellular carcinoma
TCGA-LIHC: The Cancer Genome Atlas – Liver HCC
SOCS: Suppressor of cytokine signaling
