# Supplementary material for: Prognostic significance of SOCS1 and SOCS3 tumor suppressors and oncogenic signaling pathway genes in hepatocellular carcinoma
Source: BMC Cancer. 2020 Aug 17;20:774. doi: 10.1186/s12885-020-07285-3 (PMC7433106; doi:10.1186/s12885-020-07285-3)
Supplement: Supplementary file 1 — Additional file 1: Figure S1. Workflow of this study. Figure S2. Expression of oncogenic signaling pathway genes that synergize with SOCS1 or SOCS3 in predicting prognosis by the Cox proportional harzards model in the murine models. DAB2 and CXCL8 synergize with SOCS1 or SOCS3 in predicting prognosis by the Cox proportional harzards model (shown in Table 2). As the CXCL8 (IL-8) gene is not present in the mouse, we examined the genes coding for mouse chemokines KC (Cxcl1), MIP-2 (Cxcl2) and LIX (Cxcl2), which are considered the functional equivalent of human CXCL8 in promoting neutrophil migration.(A) Partial hepatectomy was carried out on 8–10 weeks old mice lacking Socs1 or Socs3 in hepatocytes and control mice. The expression of the indicated genes in the regenerating livers was evaluated 24 h later by qRT-PCR. n = 4–6 mice per group. (B) Mice lacking Socs1 or Socs3 in hepatocytes and control mice were treated with DEN (25 mg/kg body weight) at 2 weeks of age and livers collected at 8–10 months of age. Tumor nodules and adjacent normal liver tissues were resected and expression of the indicated genes was evaluated by qRT-PCR. n = 4–6 mice per group. p-values were calculated by one-way ANOVA along with Tukey’s Multiple Comparison test: * p < 0.0001. Table S1. List of qRT-PCR primers used in this study. Table S2. Impact of the expression of oncogenic pathway genes on survival probability in the TCGA-LIHC dataset. Table S3. Combinations of high SOCS1 or high SOCS3 and oncogenic signalling pathway genes that show significant prognosis in the Cox proportional hazard model. [file 12885_2020_7285_MOESM1_ESM.pdf]

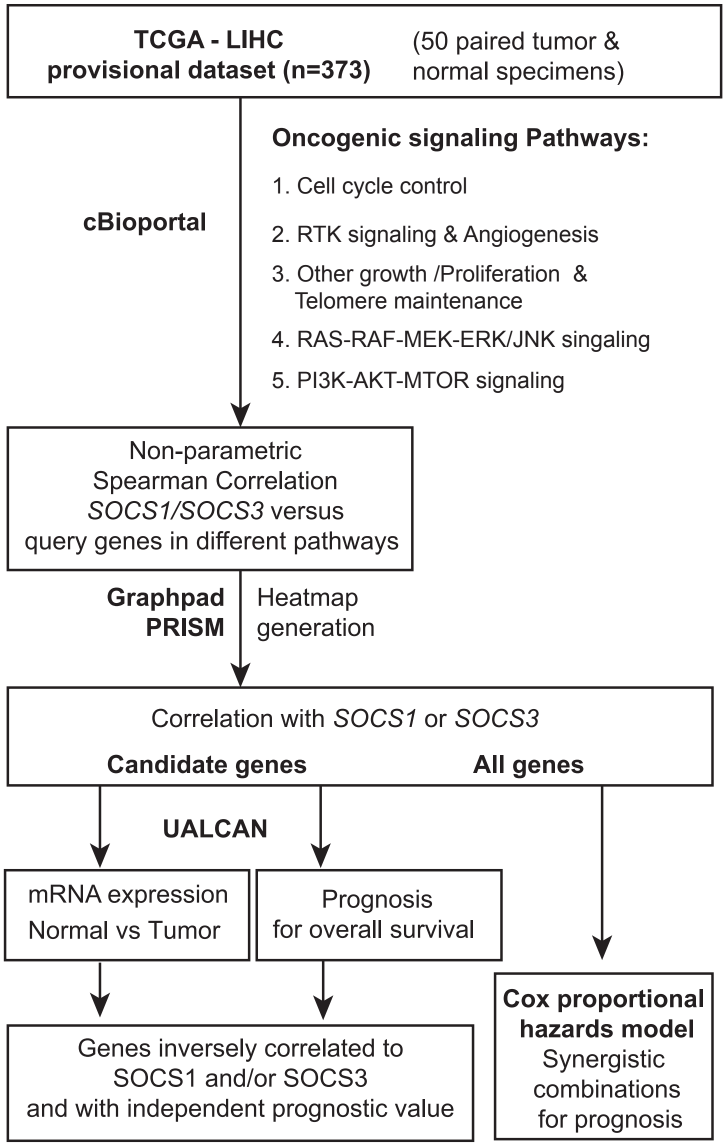

Figure S1. Workflow of this study.

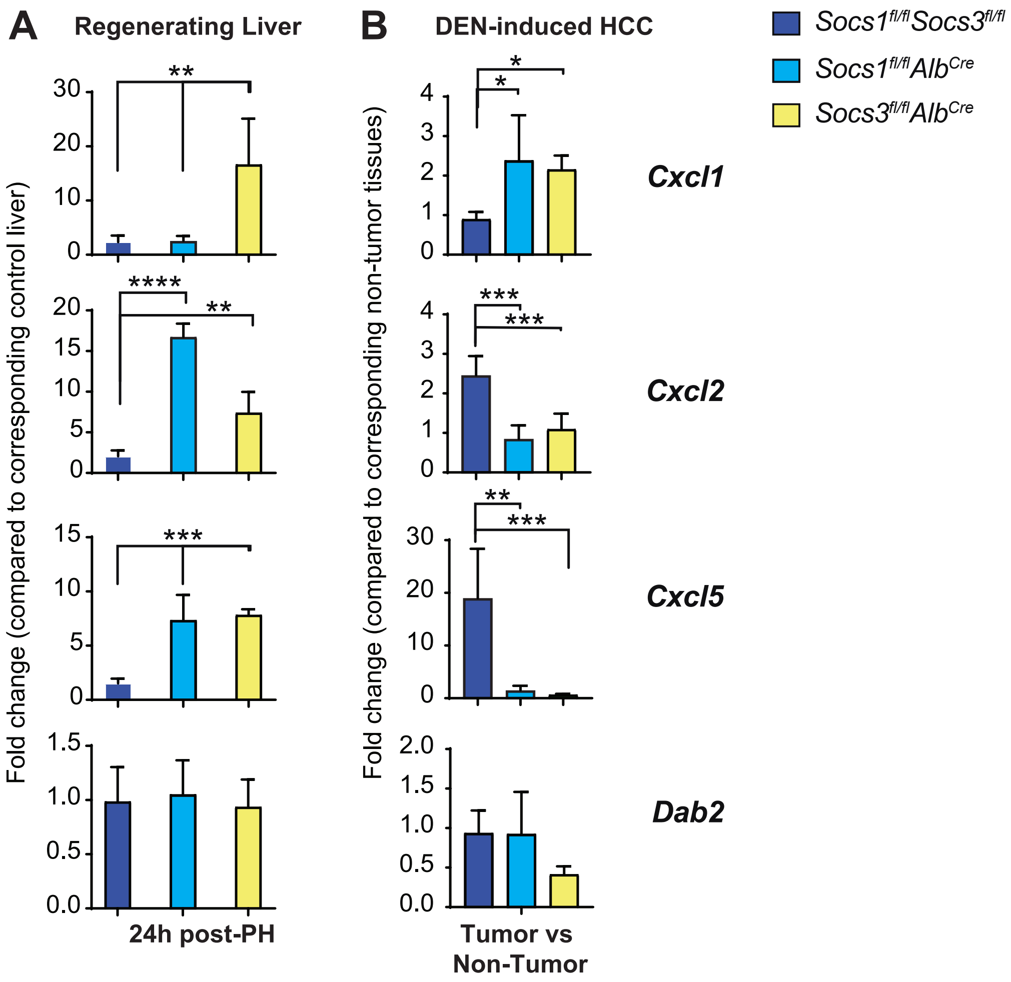

Figure S2. Expression of oncogenic signaling pathway genes that synergize with *SOCS1* or *SOCS3* in predicting prognosis by the Cox proportional harzards model in the murine models. *DAB2* and *CXCL8* synergize with *SOCS1* or *SOCS3* in predicting prognosis by the Cox proportional harzards model (shown in Table 2). As the *CXCL8* (IL-8) gene is not present in the mouse, we examined the genes coding for mouse chemokines KC (*Cxcl1*), MIP-2 (*Cxcl2*) and LIX (*Cxcl2*), which are considered the functional equivalent of human CXCL8 in promoting neutrophil migration.(A) Partial hepatectomy was carried out on 8-10 weeks old mice lacking *Socs1* or *Socs3* in hepatocytes and control mice. The expression of the indicated genes in the regenerating livers was evaluated 24h later by qRT-PCR. n=4-6 mice per group. (B) Mice lacking *Socs1* or *Socs3* in hepatocytes and control mice were treated with DEN (25 mg/kg body weight) at 2 weeks of age and livers collected at 8-10 months of age. Tumor nodules and adjacent normal liver tissues were resected and expression of the indicated genes was evaluated by qRT-PCR. n=4-6 mice per group. p-values were calculated by one-way ANOVA along with Tukey's Multiple Comparison test: * $p < 0.05$, ** $p < 0.01$, *** $p < 0.001$, **** $p < 0.0001$.

Supplementary Table S1: List of qRT-PCR primers used in this study.

Gene name	Gene ID	Sense primer	Anti-sense primer	Amplicon Size (bp)
Aurka	NM_011497	TGAGTTGGAAAGGGACATGG	GGACCCCTCTGCATTGATAAG	136
Cdk2	NM_016756	GCATTCCTCTTCCCCTCATC	GGACCCCTCTGCATTGATAAG	128
Mlst8	NM_019988	CTGAGTCTTCCATCACGTCTG	GATCTTGGTCTTAGGGATGAGC	143
Rptor	NM_028898	CACTCCTTGTCTTCATCTGGG	TGTCATGGTCCTATGTTCAGC	140
Map3k4	NM_011948	ATGTTGGACTGAGGAAGGTG	TGGTCGTTAGGCTGAAATCG	145
36B4 (Rplp0)	NM_007475.5	TCTGGAGGGTGTCCGCAA	CTTGACCTTTTCAGTAAGTGG	154
Dab2	NM_001037905	AGCCAGTCTTTTGCTCATCTG	TCCAAAGGGTTCAGCTCATC	140
Cxcl1	NM_008176.3	AACCGAAGTCATAGCCACAC	CAGACGGTGCCATCAGAG	147
Cxcl2	NM_009140.2	AAAATCATCCAAAAGATACTGAACAA	CTTTGGTTCTTCCGTTGAGG	91
Cxcl5	NM_009141.3	GGGAAACCATTGTCCCTGA	TCCGATAGTGTGACAGATAGGAAA	92

Supplementary Table S2. Impact of the expression of oncogenic pathway genes on survival probability in the TCGA-LIHC dataset.

Oncogenic signaling pathway	Gene Name*	High exp. (n)	Low/Med. Exp (n)	Survival probability p-value	Expression Level for poor prognosis
**Cell Cycle**	*STAT5B*	92	273	0.96	
	CDK6	90	275	0.22	
	RBL2	93	272	0.76	
	CDK2	90	275	**0.011**	High
	CCND1	90	275	0.95	
	CDKN1B	92	273	0.18	
	RB1	90	275	0.077	
	RBL1	91	274	**0.044**	High
	E2F7	92	273	**0.0044**	High
	E2F8	91	274	**0.006**	High
	JAK1	91	274	0.17	
	E2F6	89	276	**0.00021**	High
	MYC	91	274	0.32	
	E2F1	92	273	0.1	
	CDK1	89	276	**<0.0001**	High
	CDC25A	89	276	**<0.0001**	High
	CDKN2B	91	274	**0.0016**	High
	E2F5	89	276	**0.0021**	High
	CCNB1	90	275	**<0.0001**	High
	CDK4	88	277	**<0.0001**	High
	SRC	92	273	**0.00049**	High
	CDKN2A	91	274	**0.014**	High
	STAT2	91	274	0.49	
	CCNA1	92	273	0.33	
	E2F3	89	276	0.13	
	E2F2	90	275	0.096	
	E2F4	88	277	**0.00019**	High
	CCNE1	92	273	**0.00077**	High
	STAT1	91	274	0.16	
	CDKN1A	91	274	0.75	
	JAK2	90	275	0.91	
	STAT5A	91	274	0.75	
	STAT3	92	273	0.42	
	CCND2	90	275	0.66	
**RTK signaling & Angiogenesis**	*MET*	93	272	0.64	
	ERBB2	93	272	0.34	
	KDR	93	272	0.088	
	EGFR	93	272	0.54	
	VEGFA	90	275	**<0.0001**	High
	KIT	91	274	0.093	
	PDGFB	92	273	0.96	
	IGF1	93	272	0.089	

	PDGFRB	92	273	0.35	
	ERBB4	92	273	0.67	
	ERBB3	92	273	**0.0081**	High
	PDGFA	92	273	0.62	
	FGF1	92	273	0.46	
	IGF1R	89	276	0.29	
	FGFR1	92	273	0.44	
	PDGFRA	91	274	0.24	
	VEGFB	91	274	0.61	
	CXCR1	91	274	0.52	
	CXCR2	91	274	0.96	
	CXCL8	92	273	**0.01**	High
**Other growth & Proliferation/TERT**	*DPH1*	92	273	0.67	
	DLEC1	92	273	0.24	
	AURKA	90	275	**0.0016**	High
	IGF1	93	272	0.089	
	OPCML	92	273	0.7	
	FGF1	92	273	0.46	
	PLAGL1	89	276	0.74	
	IGF1R	89	276	0.29	
	FGFR1	92	273	0.44	
	CSF1	91	274	**<0.0001**	High
	CSF1R	90	275	0.15	
	TERT	91	274	0.51	
	TERC	92	273	0.31	
**RAS-RAF-MEK-ERK/JNK**	***MAPK1***	91	274	**0.013**	High
	MAPK8	93	272	0.13	
	MAPK14	91	274	0.19	
	MAPK6	90	275	0.14	
	MAP2K5	92	273	0.93	
	BRAF	92	273	**0.0066**	High
	RAF1	91	274	0.17	
	MAP3K2	93	272	0.81	
	MAP3K4	89	276	**0.0025**	High
	KRAS	93	272	0.11	
	MAP2K4	92	273	0.51	
	MAP3K1	90	275	0.28	
	MAPK9	90	275	0.21	
	MAPK4	93	272	0.56	
	MAP3K3	92	273	0.14	
	MAP2K1	91	274	0.28	
	MAP2K3	91	274	0.22	
	MAPK7	91	274	**0.0061**	High
	MAP2K2	90	275	**0.0034**	High
	RAB25	93	272	0.51	
	MAPK12	92	273	**0.026**	High
	MAPK3	92	273	**0.00015**	High

	HRAS	88	277	**0.00073**	High
	RASSF1	89	276	**0.013**	High
	MAP3K5	92	273	0.54	
	DAB2	92	273	**0.00046**	High
**PI3K-AKT-MTOR**	***PIK3R1***	93	272	**0.012**	Low
	PDPK1	93	272	0.25	
	RPTOR	90	275	**0.0043**	High
	PTEN	91	274	0.47	
	AKT2	92	273	0.38	
	PIK3CA	93	272	**0.0088**	High
	MTOR	93	272	0.18	
	TSC1	90	275	**0.0028**	High
	TSC2	92	273	0.2	
	FOXO1	93	272	**0.027**	Low
	RICTOR	92	273	**0.0068**	High
	FOXO3	91	274	**0.0083**	High
	RHEB	92	273	**0.0026**	High
	PIK3R2	89	276	**0.018**	High
	MLST8	89	276	**0.0014**	High
	AKT1S1	91	274	**0.00073**	High
	AKT1	90	275	**0.0015**	High

* Gene names in **boldface**: Significant correlation with *SOCS1* (vary with *SOCS3*; see figures 3 to 6). Color codes:
Green: negative correlation (mutual exclusivity) with SOCS1 or SOCS3.
Black: no correlation with SOCS1.
Red: positive correlation (co-occurrence) with SOCS1.
Orange: Prognosis discordant from expected functions.

Supplementary Table S3. Combinations of high *SOCS1* or high *SOCS3* and oncogenic signalling pathway genes that show significant prognosis in the Cox proportional hazard model.

Selected Combinations	Oncogenic signaling Pathway	Multivariate Cox model p-value	Univariate log-rank p-value	Survival probability	Number of subjects	HR [95% confidence intervals]
High *SOCS1* + low *E2F5*	Cell cycle	0.0051	0.0051	Better	22	0.07 [0.01 - 0.05]
High *SOCS1* + High *PDGFA*	RTK signalling, angiogenesis	0.0241	0.0323	Better	26	0.23 [0.09 - 0.61]
High *SOCS1* + High *KDR*	RTK signalling, angiogenesis	0.0219	0.0414	Better	19	0.28 [0.09 - 0.89]
High *SOCS1* + High *E2F7*	Cell cycle	0.0012	0.0334	Poor	21	4.98 [2.29 - 10.84]
High *SOCS1* + Low *OPCML*	Proliferation	0.0304	0.0483	Poor	23	2.45 [1.34 - 4.49]
High *SOCS3* + High *STAT5B*	Cell cycle	0.008	0.0156	Better	15	0.11 [0.02 - 0.79]
High *SOCS3* + Low *PDGFB*	RTK signalling, angiogenesis	0.0394	0.0317	Better	13	0.16 [0.02 - 1.17]
High *SOCS3* + High *FOXO1*	PI3K-AKT pathway	0.0214	0.0248	Better	34	0.42 [0.2 - 0.92]
High *SOCS3* + High *CDK1*	Cell cycle	0.0001	0.0001	Poor	19	4.05 [2.15 - 7.63]
High *SOCS3* + High *AURKA*	Proliferation	0.0109	0.0109	Poor	20	2.48 [1.29 - 4.77]
High *SOCS3* + Low *MAP2K4*	MAPK pathway	0.0102	0.0143	Poor	16	2.65 [1.41 - 4.96]

Limitations: Comparison of the top or bottom 25% (high or low) with the rest (75) is arbitrary. Some combination groups have only a very few number of cases (Total n=362).
Color codes for high/low expression of individual genes: Red: Poor prognosis; Green: Good prognosis; Blue: No prognostic value. *, ** synergy with both low *SOCS1* and low *SOCS3*.
